# Improving the Oxidative Stability of a High Redox Potential Fungal Peroxidase by Rational Design

**DOI:** 10.1371/journal.pone.0124750

**Published:** 2015-04-29

**Authors:** Verónica Sáez-Jiménez, Sandra Acebes, Victor Guallar, Angel T. Martínez, Francisco J. Ruiz-Dueñas

**Affiliations:** 1 Centro de Investigaciones Biológicas, Consejo Superior de Investigaciones Científicas, Madrid, Spain; 2 Joint Barcelona Supercomputing Center—Centre for Genomic Regulation, Institute for Research in Biomedicine Research Program in Computational Biology, Barcelona Supercomputing Center, Barcelona, Spain; Universidade Nova de Lisboa, PORTUGAL

## Abstract

Ligninolytic peroxidases are enzymes of biotechnological interest due to their ability to oxidize high redox potential aromatic compounds, including the recalcitrant lignin polymer. However, different obstacles prevent their use in industrial and environmental applications, including low stability towards their natural oxidizing-substrate H_2_O_2_. In this work, versatile peroxidase was taken as a model ligninolytic peroxidase, its oxidative inactivation by H_2_O_2_ was studied and different strategies were evaluated with the aim of improving H_2_O_2_ stability. Oxidation of the methionine residues was produced during enzyme inactivation by H_2_O_2_ excess. Substitution of these residues, located near the heme cofactor and the catalytic tryptophan, rendered a variant with a 7.8-fold decreased oxidative inactivation rate. A second strategy consisted in mutating two residues (Thr45 and Ile103) near the catalytic distal histidine with the aim of modifying the reactivity of the enzyme with H_2_O_2_. The T45A/I103T variant showed a 2.9-fold slower reaction rate with H_2_O_2_ and 2.8-fold enhanced oxidative stability. Finally, both strategies were combined in the T45A/I103T/M152F/M262F/M265L variant, whose stability in the presence of H_2_O_2_ was improved 11.7-fold. This variant showed an increased half-life, over 30 min compared with 3.4 min of the native enzyme, under an excess of 2000 equivalents of H_2_O_2_. Interestingly, the stability improvement achieved was related with slower formation, subsequent stabilization and slower bleaching of the enzyme Compound III, a peroxidase intermediate that is not part of the catalytic cycle and leads to the inactivation of the enzyme.

## Introduction

**Fig 1:**
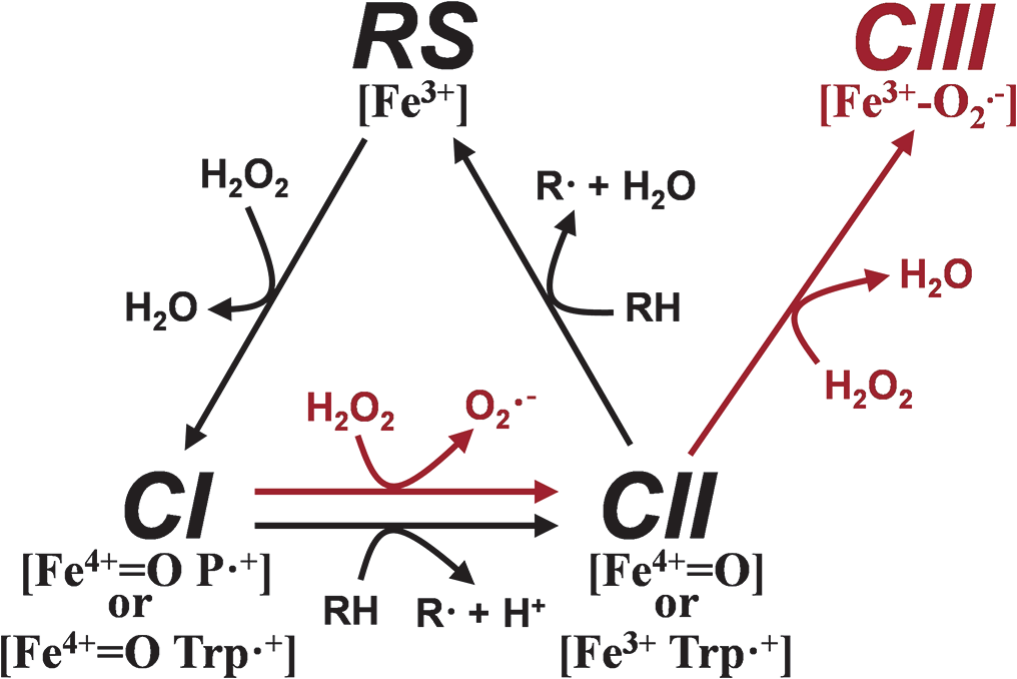
Versatile peroxidase catalytic cycle (black arrows) and CIII formation by hydrogen peroxide (red arrows). RS, resting state peroxidase containing Fe^3+^ heme; CI, compound I retaining two oxidizing equivalents in the form of a Fe^4+^ = O and porphyrin cation radical, P·^+^, or Fe^4+^ = O and tryptophanyl cation radical, Trp·^+^ (on Trp164); CII, compound II bearing only one oxidation equivalent on the Fe^4+^ = O or Trp·^+^; CIII, compound III containing Fe^3+^ and superoxide anion radical, O_2_^.-^; and RH, reducing substrates of CI and CII.

Lignin removal is a key step for carbon recycling in terrestrial ecosystems, as well as a central issue for industrial utilization of plant biomass [1]. In nature, white-rot fungi are the main organisms responsible for lignin biodegradation in a process that has been described as an enzymatic combustion [2]. These fungi have developed an extracellular ligninolytic machinery made up of oxidoreductases including peroxidases, laccases and oxidases, among others [3]. Manganese peroxidases (MnP, E.C: 1.11.1.13), lignin peroxidases (LiP, E.C. 1.11.1.14), and versatile peroxidases (VP, E.C.1.11.1.16) forming part of this machinery are unique to white-rot basidiomycetes [4] although they can be absent from some atypical or transitional species [5]. VP shares catalytic properties with MnP and LiP, and also with non-ligninolytic generic peroxidases (GP, E.C. 1.11.1.7), due to combination of the catalytic sites characterizing these enzymes [6]. This results in a high redox potential peroxidase with a wide substrate specificity, able to oxidize Mn^2+^ [7] and high and low redox potential compounds [8–10]. Its catalytic promiscuity makes VP a biocatalyst of industrial and environmental interest. The use of this enzyme has been considered as an interesting possibility in lignin transformation and delignification processes [11–13]. Its ability to transform polycyclic aromatic hydrocarbons, phenolic and non-phenolic aromatic pollutants, pesticides and industrial dyes has been confirmed [14, 15]. Moreover, it has been also proposed as a valuable tool for generating new biomolecules [16]. However, the potential of this and the other high redox potential peroxidases cannot be exploited due to different obstacles that prevent their utilization at industrial level [1]. Of these, perhaps the most surprising is the inactivation by peroxide excess, since this compound is the substrate strictly necessary for enzyme activation. This process has been described as a suicide inactivation related to the formation of Compound III (CIII), a peroxidase intermediate that is not part of the catalytic cycle but can lead to enzyme inactivation [17–19]. The VP catalytic cycle (Fig 1, black arrows) [10] is initiated by the reaction of the enzyme resting state (RS, containing Fe^3+^ heme) with one molecule of H_2_O_2_ in a two-electron reaction yielding Compound I (CI). This transient state contains two oxidation equivalents, one as a ferryl-oxo iron (Fe^4+^ = O) and the other delocalized as a porphyrin radical or as a tryptophanyl radical (on Trp164). CI catalyzes the one-electron substrate oxidation in direct contact with heme or through the tryptophanyl radical forming Compound II (CII). This intermediate retains the Fe^4+^ = O state (after porphyrin or tryptophanyl radical reduction) or transfers the remaining oxidation equivalent to the tryptophan residue as in CI. Finally, CII can also produce one-electron oxidization of substrates directly in contact with the heme or through the tryptophanyl radical restoring the enzyme resting state. Both CI and CII are very reactive intermediates that, in the absence of a normal reducing substrate and in the presence of high H_2_O_2_ concentration, are finally converted to CIII, a superoxide anion (O_2_^.-^) containing Fe^3+^ species (Fig 1, red arrows) [17]. Once formed, CIII can follow different decomposition pathways under excess of H_2_O_2_ generating reactive oxygen species able to oxidize the porphyrin moiety or amino acid side chains (such as those of methionines) leading to enzyme inactivation [19].

Taking into account this information, we addressed the oxidative stability improvement in VP using different strategies consisting in: i) substituting methionines for less-oxidizable residues to prevent or minimize inactivation by formation of methionine derivatives; ii) modifying the environment of the histidine residue involved in the two-electron activation of the resting enzyme to affect the stability/reactivity of CI and CII, as CIII precursors; and iii) combining the above two strategies. In the light of the results obtained, a rational explanation is proposed for the observed oxidative stability improvements.

## Materials and Methods

### Chemicals

Isopropyl-β-D-thiogalactopyranoside (IPTG), dithiothreitol, hemin, oxidized glutathione, veratryl alcohol (VA), manganese(II) sulphate and sodium tartrate were from Sigma-Aldrich; urea and H_2_O_2_ from Merck; and 2,2'-azino-bis(3-ethylbenzothiazoline-6-sulfonate) (ABTS) from Roche.

### Amino acid analysis

The amino acid composition of the native VP incubated in the absence and in the presence of a 5000-fold stoichiometric excess of H_2_O_2_, at 25°C for 40 min, was determined with a Biochrom 30 amino acid analyzer. Previous to the amino acid analysis, the samples (24 μg of protein) were subjected to an acid hydrolysis (6 N HCl) for 24 h under vacuum. The H_2_O_2_ concentration in this and other experiments was determined using ε_240_ = 39.4 M^-1^ cm^-1^ [20].

### Design of mutated variants at the heme distal side

Residues in the contact interface between helixes D and B, the latter containing catalytic Arg43 and His47 were identified. Thr45 and Ile103 located immediately on the catalytic amino acid residues in helixes B and D, respectively, were selected for site-directed mutagenesis. Mutations at these two residues sought to reduce the distance between the two helices. Alternative amino acid residues at these positions were checked *in silico* by using the Swiss-Pdb Viewer mutation tool. Acidic, basic and easily oxidizable amino acids as well as glycine and residues with side chains larger than those of threonine and isoleucine were excluded from the analysis. Amino acid residues with their rotamers exhibiting the lowest score were selected for subsequent site directed mutagenesis.

### Site-directed mutagenesis

All VP variants were produced using the QuikChange Site-Directed Mutagenesis kit from Stratagene. Each mutation was introduced by PCR using the expression vector pFLAG1 (International Biotechnologies Inc.) harbouring the mature protein-coding sequence of *Pleurotus eryngii* VP (allelic variant VPL2; GenBank AF007222) (named pFLAG1-VPL2) [21] as template. For each mutation, both a direct and a reverse primer were designed complementary to opposite strands of the same DNA region containing the desired mutation. Only the direct sequences with indication of the changed triplets (underlined) and the mutations introduced (bold) are listed below: (i) M152V, 5'-CC ATT CTT GCC AGA **G**TG GGT GAC GCA GGC-3'; (ii) M247F, 5'-GCC TGC GAA TGG CAG TCC **T**T**C** GTT AAC AAC CAA CCG-3'; (iii) M262F, 5'-C CGT TTC GCT GCT ACC **T**T**C** TCG AAG ATG GCT CTT CTT GGC C-3'; (iv) M265L, 5'-GCT ACC ATG TCG AAG **T**TG GCT CTT CTT GGC C-3'); (v) M262F/M265L, 5'-CGT TTC GCT GCT ACC **T**T**C** TCG AAG **T**TG GCT CTT CTT GGC C-3'; (vi) M152F/M262F/M265L was obtained using pFLAG1-VPL2-M262F/M265L as template and primers for the M152F mutation, 5'-CC ATT CTT GCC AGA **T**T**C** GGT GAC GCA GGC-3'; (vii) M152F/M247F/M262F/M265L was obtained using pFLAG1-VPL2-M152F/M262F/M265L as template and the M247F primers; (viii) T45A/I103T was obtained using pFLAG1-VPL2 as template and the T45A, 5'-C GAG TCC CTT CGT TTG **G**CT TTC CAC GAT GCA ATC GG-3', and I103T, 5'-CC GCC GGC GAC TTC A**C**T CAA TTT GCT GGC GCC G-3', primers in two consecutive PCR reactions; and (ix) T45A/I103T/M152F/M262F/M265L was obtained using pFLAG1-VPL2-M152F/M262F/M265L as template and the T45A and I103T primers in two successive PCR amplifications.

PCR reactions were carried out in an Eppendorf Mastercycler Pro S using 10 ng of template DNA, 250 μM each dNTP, 125 ng of direct and reverse primers, 2.5 units of *Pfu* Turbo DNA polymerase AD (Stratagene) and the manufacture´s reaction buffer. Reaction conditions were as follows: (i) a “hot start” of 95°C for 1 min; (ii) 18 cycles at 95°C for 50 s, 55°C for 50 s, and 68°C for 10 min; and (iii) a final cycle at 68°C for 10 min. The mutated sequences were confirmed by DNA sequencing using an ABI 3730 DNA Analyzer (Applied Biosystem). pFLAG1-VPL2 plasmids containing mutations described above were transformed into *Escherichia coli* DH5α for propagation

### Enzyme production, activation and purification

Native recombinant VP and its site-directed variants were expressed in *E*. *coli* W3110. Cells were grown in Terrific Broth [22] until OD_500_~1 (~3h). Then protein expression was induced with 1 mM IPTG and cells were grown for a further 4 h. The apoenzyme accumulated in inclusion bodies and was recovered by solubilization in 50 mM Tris-HCl (pH 8.0) containing 8 M urea, 1 mM EDTA, and 1 mM dithiothreitol for 30 min at room temperature. The subsequent *in vitro* folding of the solubilised apoprotein and purification of the active enzyme were performed as described by Pérez-Boada et al. [21]. Enzyme concentration was determined from the absorbance of the Soret band (ε_407_ = 150 mM^-1^ cm^-1^) [23].

### Steady-state kinetics

Oxidation of VA (veratraldehyde ε_310_ = 9300 M^-1^ cm^-1^) was estimated at pH 3.0; that of ABTS (cation radical ε_436_ = 29300 M^-1^ cm^-1^) at pH 3.5 and that of Mn^2+^ (Mn^3+^-tartrate complex ε_238_ = 6500 M^-1^ cm^-1^) at pH 5.0. All enzymatic activities were measured as initial velocities from linear increments of absorbance due to the appearance of the reaction product. Reactions were performed in 0.1 M sodium tartrate and 0.1 mM H_2_O_2,_ at 25°C, and followed using a Shimadzu UV-1800 spectrophotometer. Means and standard errors for apparent affinity constant (Michaelis constant, *K*_m_) and enzyme turnover (catalytic constant, *k*_cat_) were obtained by non-linear least-squares fitting of the experimental measurements to the Michaelis-Menten model. Fitting of these constants to the normalized equation *υ* = (*k*_cat_/*K*_m_)[S]/(1+[S]/*K*_m_) yielded the catalytic efficiency values (*k*_cat_/*K*_m_) with their standard errors.

### Transient-state kinetics

VP CI formation was measured at 25°C using a stopped-flow equipment (Bio-Logic) including a three-syringe module (SFM300) synchronized with a diode array detector (J&M), and Bio-Kine software. The resting enzyme (1 μM final concentration) was mixed with increasing concentrations of H_2_O_2_ in 0.1 M sodium tartrate (pH 3.0) and the reaction was followed at 397 nm (isosbestic point of VP CI and CII). All kinetic traces exhibited single-exponential character from which pseudo first-order rate constants were calculated.

### Oxidative stability studies

The oxidative stability was determined by enzyme incubation in a wide range of H_2_O_2_:VP molar ratios in 10 mM sodium tartrate, pH 5.0. The time course of oxidative inactivation was followed by measuring the residual activity of the enzyme (0.01 μM) as the oxidation of 6 mM Mn^2+^ (saturating conditions) as described above. The enzyme incubated under the same experimental conditions in absence of H_2_O_2_ was used as a reference. The experimental data of residual activity *vs* time at each H_2_O_2_:VP ratio were fitted to an exponential decay model and a pseudo first-order inactivation rate constant (*k*_obs_, s^-1^) was obtained. The *k*_obs_ values for each VP variant at all the H_2_O_2_:VP ratios tested were fitted to a linear or a sigmoidal (Hill equation) model. From this fitting an apparent second-order inactivation rate constant (*k*_app_, s^-1^·M^-1^) (for linear fits), and a first-order inactivation rate constant (*k*_i_) and the H_2_O_2_:VP molar ratio resulting in the half maximal inactivation rate (*K*_I_) (for sigmoid fits) were calculated. Fitting of *k*_i_ and *K*_I_ to the normalized equation *k*_obs_ = (*k*_i_/*K*_I_) *K*_I_ R^n^/(*K*_I_^n^+R^n^) (where n is the Hill coefficient and R is the H_2_O_2_:VP molar ratio) yielded the *k*_app_ values (*k*_i_/*K*_I_) with their corresponding standard errors.

### Spectral analysis of transient states in H_2_O_2_ reactions

Spectral analyses of native VP and mutated variants (10 μM) were carried out in a millisecond to second time scale after activating the enzyme with a 5000-fold stoichiometric excess of H_2_O_2_ in 0.1 M sodium tartrate (pH 5.0), at 25°C. The spectra were obtained using the stopped-flow equipment described above. The rate of CIII formation was determined at a wavelength (581 nm) at which the change in absorbance was specific for this reaction. Subsequent bleaching of 5 μM CIII, generated as previously described, was followed at its Soret maximum (419 nm) in a minute time scale using an Agilent 8454 diode array spectrophotometer. Conversion of the native VP and the T45A/I103T/M152F/M262F/M265L variant CIII to RS was measured after removing the excess of H_2_O_2_ by incubation with catalase (75 mg/ml) for 1 min at 25°C. Afterwards, 50 equivalents of Mn^2+^ were added to the reaction and the conversion to RS followed at 407 nm (Soret band). CIII formation, enzyme bleaching and CIII conversion to RS exhibited a single-exponential behaviour from which pseudo first-order rate constants (*k*_obs_, s^-1^) were calculated.

## Results

### Strategies to improve the VP oxidative stability and kinetic analysis of the designed variants

The oxidative stability of VP was investigated by enzyme incubation with increasing stoichiometric excesses of H_2_O_2_. The enzyme residual activity was measured over time, observing progressive inactivation at all the H_2_O_2_:VP molar ratios assayed (from 500:1 to 40000:1). The amino acid composition analysis of the inactivated enzyme at a 5000:1 H_2_O_2_:VP ratio, after 40 min incubation at 25°C, revealed that methionine residues were oxidized to methionine sulfone (see S1 Information on oxidative inactivation).

**Fig 2:**
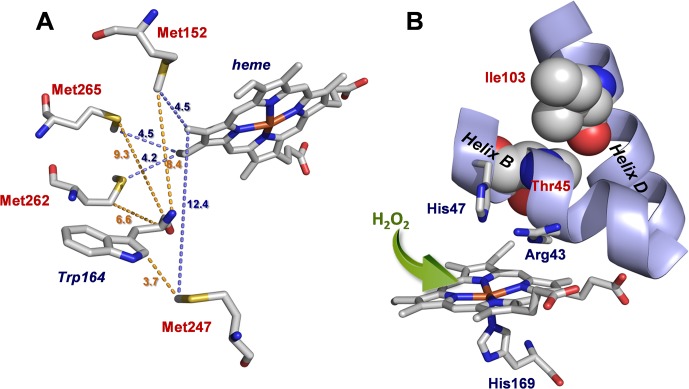
Details of native VP crystal structure (PDB entry 2BOQ). **A**, Methionine residues and their distances in Å to both the heme and the catalytic tryptophan (Trp164), shown as sticks; and **B**, distal histidine (His47) environment, including heme and Arg43 (as sticks), together with residues Thr45 and Ile103 (both as van der Waals spheres) further mutated to obtain the T45A/I103T variant (proximal His169 acting as the fifth iron ligand and H_2_O_2_ gaining access to the heme are also shown). PyMOL (http://pymol.org) was used for graphical analysis and image generation.

VP contains four methionines at positions 152, 247, 262 and 265. They are buried within the enzyme molecular structure close to both the heme cofactor (at a distance between 4.2 and 12.4 Å) and the catalytic Trp164 (at a distance between 3.7 and 9.3 Å) (Fig 2A). Considering that their oxidation is likely to be related to enzyme inactivation, they were replaced with less oxidizable amino acids by site-directed mutagenesis in single (M152V, M247F, M262F and M265L), double (M262F/M265L) and multiple (M152F/M262F/M265L and M152F/M247F/M262F/M265L) variants. As an alternative to methionine substitution, a second strategy was designed based on the modification of the distal histidine (His47) environment through introduction of mutations at Thr45 and Ile103 residues. These two amino acids are located directly above the area occupied by His47 (Thr45 in helix B and Ile103 in helix D) (Fig 2B) which is involved, together with Arg43, in the two-electron activation of the resting enzyme by H_2_O_2_ to form CI. T45A and I103T substitutions were selected after *in silico* analysis with the aim of modifying the interaction between helices B and D. Changes in the enzyme oxidation rate by H_2_O_2_ and minimization of the inactivation rate were expected, among other effects that *a priori* could not be exactly predicted. Finally, the T45A/I103T/M152F/M262F/M265L variant, including substitution of the three methionines closer to the heme plus the two mutations located above His47 was produced, combining in a single VP molecule the two strategies previously described.

**Fig 3:**
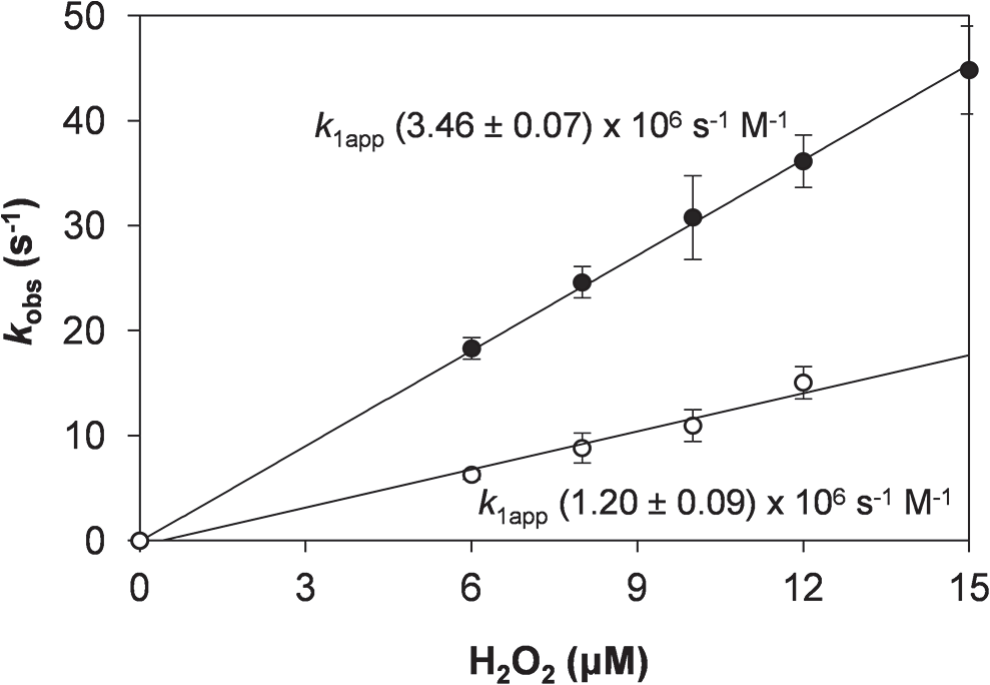
Transient-state kinetics of native VP (●) and T45A/I103T variant (○) for CI formation. Apparent second-order rate constants (*k*_1app_) expressed in s^-1^·M^-1^. Reactions were carried out at 25°C in 0.1 M sodium tartrate (pH 3.0) using 1 μM VP and increasing concentrations of H_2_O_2_. Means and 95% confidence limits are shown.

After expression and purification, the steady-state kinetic constants for oxidation of three different substrates (VA, ABTS and Mn^2+^) by native VP and the nine designed single and multiple variants were determined (see S2 Information) to know how the mutations affect the enzyme catalytic properties. In general, minor differences in catalytic efficiency were observed after replacement of one or two methionines. The rest of variants were affected in a similar way, retaining or even improving the catalytic efficiency for VA oxidation and decreasing the value of this kinetic parameter for oxidation of ABTS and Mn^2+^. Up to 16.6-fold reduction in *k*_cat_ was observed in these multiple variants for the three substrates tested. Similarly, the reaction of native VP and the different variants with H_2_O_2_ was characterized by stopped-flow spectrophotometry to know if the enzyme activation was affected by the mutations. The observed pseudo first-order rate constants (*k*_obs_) for CI formation (RS + H_2_O_2_ → CI +H_2_O) exhibited a linear dependence on H_2_O_2_ concentration, as shown in Fig 3 for native VP and the T45A/I103T variant. The apparent second-order rate constant (*k*_1app_) obtained for this variant (1.2 x 10^6^ s^-1^·M^-1^) experienced a 2.9-fold decrease compared with the native enzyme (3.46 x 10^6^ s^-1^·M^-1^).

### Oxidative stabilization

**Fig 4:**
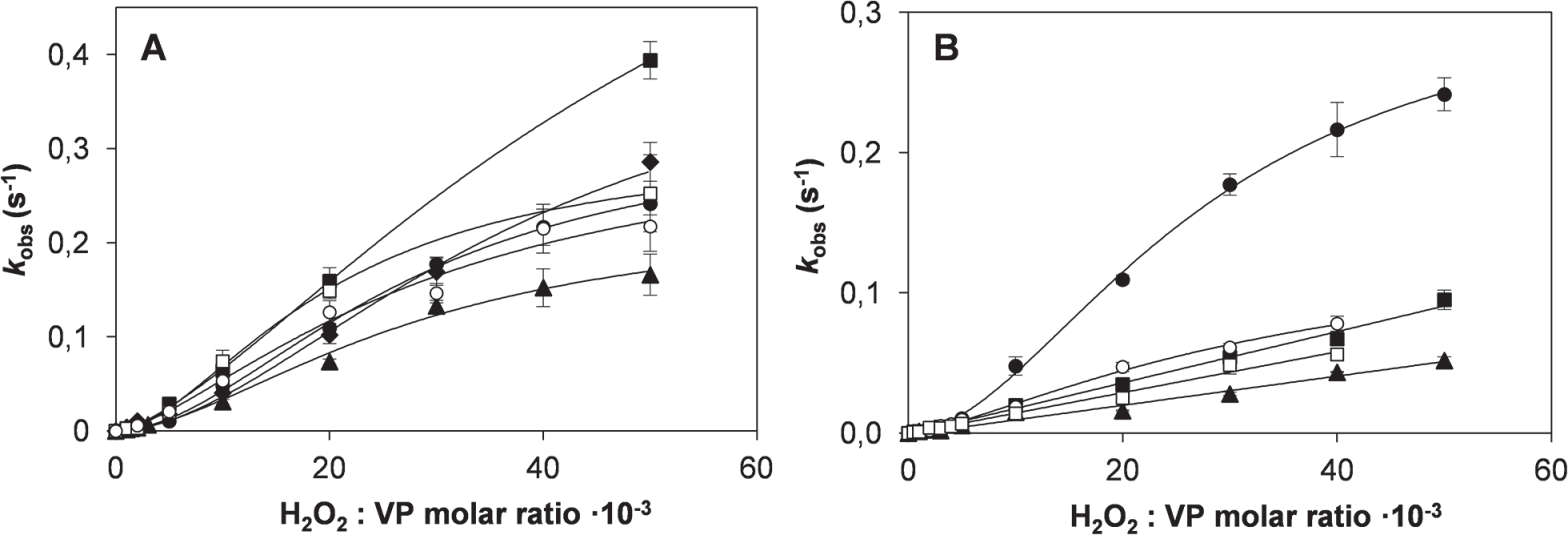
Inactivation kinetics under oxidative conditions. **A**, Native VP and single and double methionine variants: native VP (●), M247F (○), M152V (■), M265L (□), M262F (▲), M262F/M265L (♦); and **B**, native VP and multiple variants: native VP (●), T45A/I103T (○), M152F/M262F/M265L (■), M152F/M247F/M262F/M265L (□) and T45A/I103T/M152F/M262F/M265L (▲). Reactions were carried out at 25°C using 0.01 μM enzyme in 10 mM sodium tartrate (pH 5.0) in the presence of increasing H_2_O_2_ concentrations. Means and 95% confidence limits are shown.

The oxidative stability of the VP variants was compared with that of the native enzyme in the presence of increasing stoichiometric excesses of H_2_O_2_. The oxidative inactivation kinetics of the native VP followed a sigmoidal curve (Fig 4) that was fitted to a Hill equation. This enzyme is characterized by a first-order inactivation rate constant (*k*_i_) of 0.32 s^-1^, a H_2_O_2_:VP ratio resulting in the half maximal inactivation rate (*K*_I_) of 27.6 mM, an apparent second-order inactivation rate constant (*k*_app_) of 11.7 s^-1^·M^-1^ and a Hill coefficient of 1.85 (Table 1). Single and double variants at methionine residues evidenced the same sigmoidal behaviour (Fig 4A). Their inactivation kinetic constants did not substantially differ from those of the native VP, with *k*_app_ values between 8.3 and 15.5 s^-1^·M^-1^ (Table 1). Interestingly, in the triple (M152F/M262F/M265L) and quadruple (M152F/M247F/M262F/M265L) methionine variants, the inactivation kinetics fitted to a linear model (Fig 4B) instead of to a sigmoidal one, with a concomitant decrease in the *k*_app_ value (Table 1). The triple variant exhibited a *k*_app_ of 1.8 s^-1^·M^-1^, being 6.5-fold lower than the value shown by the native VP. Likewise, the quadruple variant presented a *k*_app_ of 1.5 s^-1^·M^-1^, a value slightly lower than that found for the triple variant and 7.8-fold lower compared with the native VP.

**Table 1:** Kinetic constants of the inactivation process of native VP and its mutated variants.

	*k*_i_ (s^-1^)	*K*_I_ (mM)	*k*_app_ (s^-1^·M^-1^)	n
VP	0.32 ± 0.02	27.6 ± 2.2	11.7 ± 0.2	1.85 ± 0.12
M262F	0.23 ± 0.04	27.9 ± 6.3	8.3 ± 0.5	1.73 ± 0.29
M265L	0.30 ± 0.01	20.1 ± 1.4	15.1 ± 0.5	1.75 ± 0.11
M247F	0.32 ± 0.09	29.3 ± 12.6	11.0 ± 1.7	1.35 ± 0.22
M152V	0.83 ± 0.09	53.8 ± 7.7	15.5 ± 0.5	1.46 ± 0.06
M262F/M265L	0.45 ± 0.04	38.3 ± 4.3	11.7 ± 0.4	1.80 ± 0.17
M152F/M262F/M265L	n.d.^a^	n.d. ^a^	1.8 ± 0.04	n.d. ^a^
M152F/M247F/M262F/M265L	n.d. ^a^	n.d. ^a^	1.5 ± 0.05	n.d. ^a^
T45A/I103T	0.14 ± 0.02	34.4 ± 7.8	4.1 ± 0.3	1.41 ± 0.11
T45A/I103T/M152F/M262F/M265L	n.d. ^a^	n.d. ^a^	1.0 ± 0.04	n.d. ^a^

(*k*_i_, first-order inactivation rate constant; *K*_I_, H_2_O_2_:VP molar ratio resulting in the half maximum inactivation rate; *k*_app_, apparent second-order oxidative inactivation rate constant; and n, Hill coefficient).

Reactions at 25°C in 0.1 M tartrate, pH 5, using 0.01 μM VP, final concentration, and increasing stoichiometric excesses of H_2_O_2_. Means and 95% confidence limits.

^a^n.d. Not determined because saturation was not reached.

A 2.9-fold decrease was also observed in the *k*_app_ (4.1 s^-1^·M^-1^) of the T45A/I103T variant (Table 1), mainly due to a drop in the *k*_i_ value (0.14 s^-1^), although without loss of the sigmoidal inactivation behaviour (Fig 4B). This inactivation behaviour was lost when these two mutations were combined in the T45A/I103T/M152F/M262F/M265L quintuple variant. The *k*_app_ for oxidative inactivation of this variant (1.0 s^-1^·M^-1^) was diminished to a greater extent (11.7-fold) than observed for any of the other variants.

**Fig 5:**
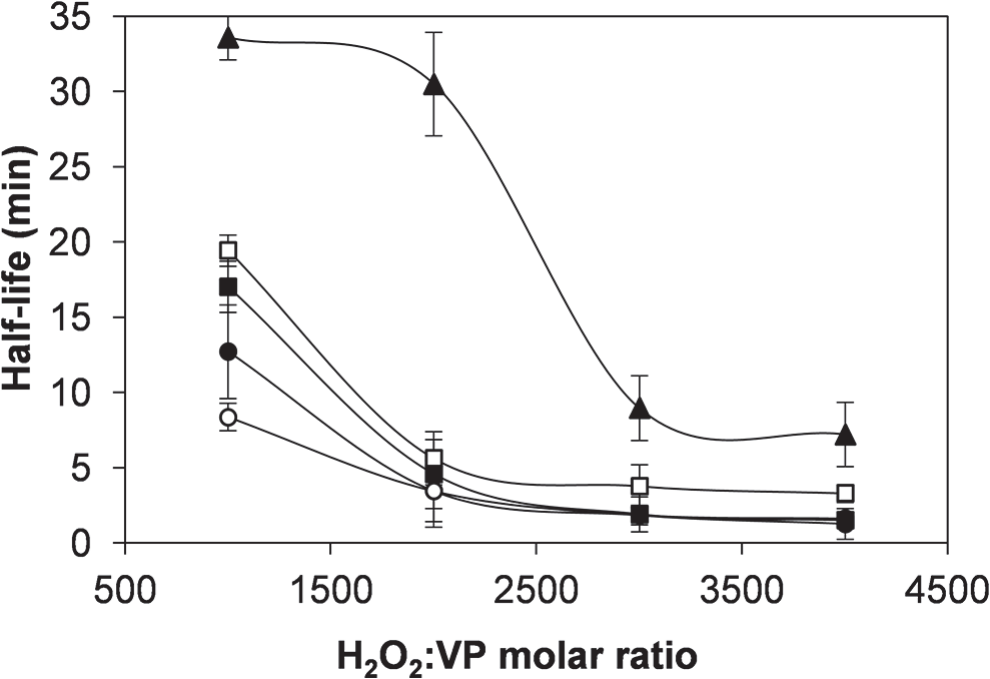
Half-life of native VP and multiple variants incubated with different H_2_O_2_:VP molar ratios. Native VP (●), T45A/I103T (○), M152F/M262F/M265L (■), M152F/M247F/M262F/M265L (□) and T45A/I103T/M152F/M262F/M265L (▲).The enzyme half-life is defined as the period of time required to inactivate the enzyme at 50% of initial activity. The residual activity of the variants (0.01 μM) was measured in 10 mM sodium tartrate (pH 5.0) using 6 mM Mn^2+^ and 0.1 mM H_2_O_2_.

Finally, the decrease in the *k*_app_ values observed in the above multiple variants could be related to the increase in the half-life of these enzymes, and this improvement was more significant at the lowest H_2_O_2_:VP ratios (Fig 5). The quintuple variant exhibited the highest half-life (over 30 min at H_2_O_2_:VP ratios of 1000:1 and 2000:1) compared with the other variants and the native VP (3.4 min at a H_2_O_2_:VP ratio of 2000:1).

### Spectral analyses

**Fig 6:**
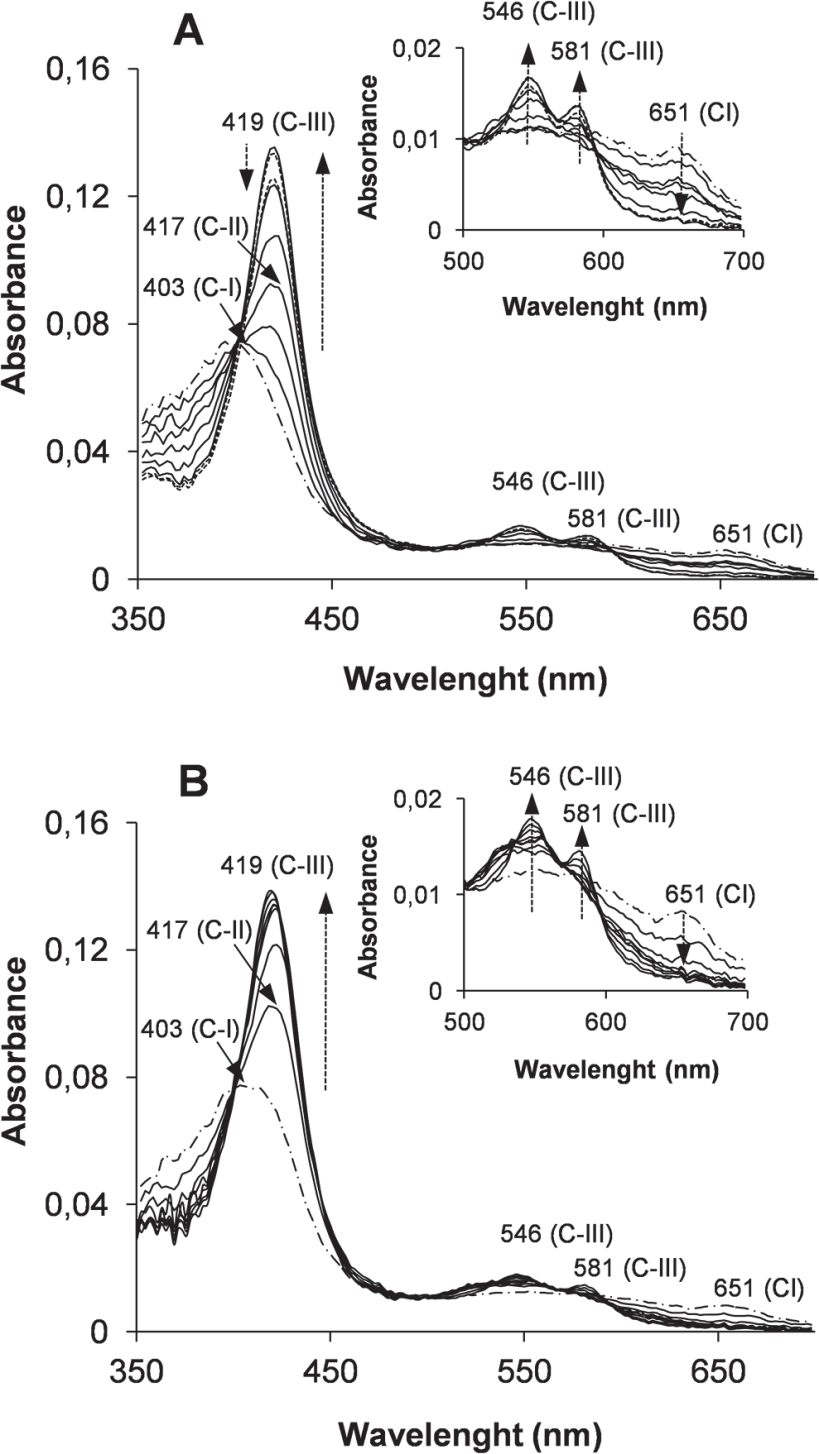
Turnover studies in the presence of excess of hydrogen peroxide. Kinetics of native VP (**A**) and its T45A/I103T/M152F/M262F/M265L variant (**B**) in the presence of a stoichiometric excess of 5000 equivalents of H_2_O_2_. Spectra were recorded at 25°C during 20 s. The dashed arrows indicate the increase and decrease of absorbance during formation of CII (with a maximum at 417 nm) and CIII (with maxima at 419 nm, 546 nm and 581 nm, and a minimum at 651 nm), and the decrease of absorbance during heme bleaching (at 419 nm). Traces correspond respectively to 0.01 s (CI spectrum both in A and B, showed as a dashed-point line); 0.1, 0.25, 0.5, 1, 2 and 5 s (continuous lines); and 10 and 20 s (continuous lines in **B**, and discontinuous lines in **A** due to heme bleaching). Details of the 500 nm-700 nm region are shown in x8 scale.

**Fig 7:**
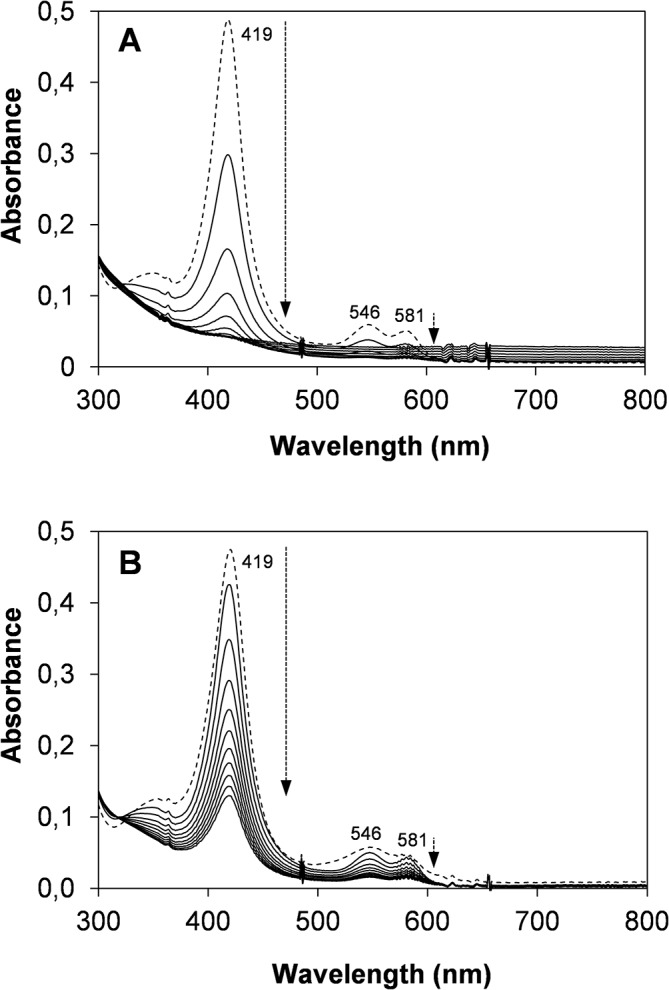
Bleaching of native VP (A) and the T45A/I103T/M152F/M262F/M265L variant (B) under high oxidative stress conditions. Both enzymes were incubated with 5000 equivalents of H_2_O_2_ at 25°C, and spectra (in the 300–800 nm range) were recorded every 2 min for 20 min. The first spectrum, obtained after 5 s of incubation, is shown as a discontinuous line and corresponds to: **A**, native VP CIII; and **B**, a mixture of CII and CIII (the latter being the major form) of the T45A/I103T/M152F/M262F/M265L variant. The arrows indicate the direction of the spectral evolution with time.

The spectral changes of the native VP and of three variants improving oxidative stability (M152F/M262F/M265L, T45A/I103T and T45A/I103T/M152F/M262F/M265L) were analyzed after enzyme activation in the presence of 5000 equivalents of H_2_O_2_ (high enough to see the transitions between the three states of the catalytic cycle in a short period of time). The first spectrum of the native enzyme, obtained at 10 ms, exhibited typical maxima of CI including the Soret band at 403 nm and the charge transfer band CT1 at 651nm (Fig 6A). Subsequently, CII was formed as observed by the displacement of the Soret band to 417 nm, the reorganization of the 500–600 nm spectral region and the disappearance of the maximum at 651 nm. Then CIII was generated exhibiting a characteristic spectrum with maxima at 419, 546 and 581 nm. Comparable spectral changes, although with different conversion rates, were obtained for the VP variants, as illustrated for the T45A/I103T/M152F/M262F/M265L variant in Fig 6B. When the reaction was extended in time (up to 20 min) strong differences in CIII decay and enzyme bleaching were observed (Fig 7), as discussed below together with the differences in CIII formation.

**Fig 8:**
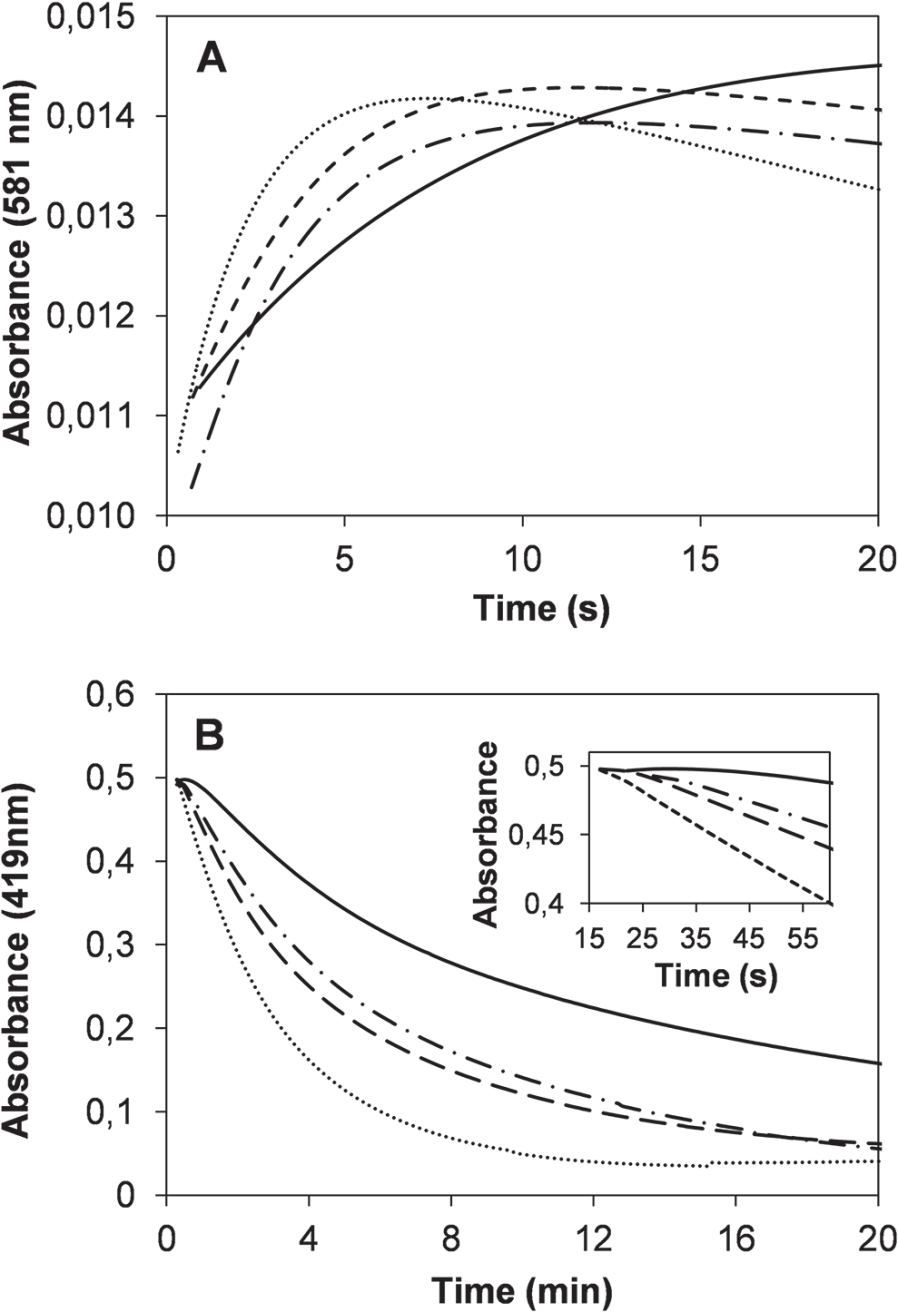
Time course of CIII formation and bleaching. **A**, CIII formation at 581nm; and **B,** CIII bleaching at 419 nm of VP (···), VP T45A/I103T (−·−), VP M152F/M262F/M265L (—-) and VP T45A/I103T/M152F/M262F/M265L (—). The variants were incubated with 5000 equivalents of H_2_O_2_ in 0.1M sodium tartrate (pH 5.0) at 25°C. The inset shows a detail of the CIII bleaching corresponding to the first min of incubation.

The rate of CIII formation (*k*_CIII(obs)_), monitored by the increase of absorbance at 581 nm (Fig 8A), was slowed down by 1.3 to 2.3-fold in the three VP variants. The T45A/I103T/M152F/M262F/M265L mutant was that exhibiting the slowest one (0.12 s^-1^ *vs* 0.27 s^-1^ for the native VP) (Table 2). Then CIII further evolved in excess of H_2_O_2_ leading to enzyme inactivation (see Table 2 for *k*_i(obs)_ values) and heme bleaching (Fig 7), which was monitored by the loss of absorbance at 419 nm (Fig 8B). The corresponding *k*_b(obs)_ values are shown in Table 2. The differences observed between both sets of constants, *k*_i(obs)_ (0.013–0.033 s^-1^) being one order of magnitude higher than *k*_b(obs)_ (0.0014–0.0058 s^-1^) for the native enzyme and mutated variants, evidenced that the loss of catalytic activity precedes heme bleaching. As previously described for CIII formation, these two events were also slowed down in the improved variants compared with the native enzyme. Native VP was very unstable and its CIII form started to be bleached from the moment it was formed (Fig 8B inset) following the spectral evolution shown in Fig 7A. By contrast, the T45A/I103T, M152F/M262F/M265L and T45A/I103T/M152F/M262F/M265L variants slowed the *k*_b(obs)_ values with respect to native VP by 1.7, 2.3 and 4.2-fold, respectively. In addition, the T45A/I103T/M152F/M262F/M265L variant remained as a stable CIII form for approximately 45 s prior to the start of heme bleaching (Fig 8B inset). Finally, CIII reversion to native VP and quintuple variant resting states were studied in the presence of Mn^2+^ as reducing substrate and similar conversion rates were obtained (0.70 and 0.75 s^-1^, respectively).

**Table 2:** Pseudo first-order rate constants for CIII formation (*k* CIII(obs)), enzyme inactivation (*k*_i(obs)_) and heme bleaching (*k*_b(obs)_) of native VP and three mutated variants in the presence of 5000 equivalents of H_2_O_2_.

	*k*_CIII(obs)_ (s^-1^)	*k*_i(obs)_ (s^-1^)	*k*_b(obs)_ (s^-1^)
VP	0.27 ± 0.05	0.033 ± 0.006	0.0058 ± 0.00002
T45A/I103T	0.21 ± 0.02	0.027 ± 0.003	0.0035 ± 0.00002
M152F/M262F/M265L	0.20 ± 0.04	0.023 ± 0.004	0.0025 ± 0.00002
T45A/I103T/M152F/M262F/M265L	0.12 ± 0.02	0.013 ± 0.002	0.0014 ± 0.00001

Reactions in 50 mM sodium tartrate, pH 5, in the presence of 5000 equivalents of H_2_O_2_. CIII formation was measured as the absorbance increase at 581 nm (Fig 8A) and heme bleaching as the absorbance decrease at 419 nm (Fig 8B). The catalytic inactivation was followed by measuring the residual activity with time, oxidizing 6 mM Mn^2+^ (saturating conditions) in the presence of 0.1 mM H_2_O_2_ and 0.01 μM enzyme. Means and 95% confidence limits.

## Discussion

H_2_O_2_ plays different roles throughout the life of heme peroxidases in general, and ligninolytic peroxidases in particular, from "birth" to "death". So, H_2_O_2_ induces the natural production of these enzymes [24, 25], is the enzyme-activating substrate, and is also involved in a mechanism-based process described as suicide inactivation [19]. The studies on *P*. *eryngii* VP, used here as a model ligninolytic peroxidase, demonstrated that this peroxidase is inactivated at increasing H_2_O_2_ concentrations in the absence of reducing substrates, confirming previous results [26, 27]. All methionines present in VP were oxidized to methionine sulfone containing larger and more polar side-chains, as also described for other peroxidases [28–30]. Considering their proximity to the heme cofactor and catalytic tryptophan, it is quite probable that a local disruption at this level causes misalignment of active site residues. This would affect electron transfer, and/or energy barriers, with an effect on the enzyme activity and stability, as shown in a variety of proteins [31]. However, although important, this is not the oxidative event that causes the complete oxidative inactivation of VP. The enzyme proceeds through the different transient states of the catalytic cycle under conditions of excess of H_2_O_2_ to reach the CIII state. This Fe^3+^-O_2_^.-^ species [17], spectrally identified here for the first time in VP, is not part of its normal catalytic cycle [10] and leads to the irreversible enzyme inactivation and heme destruction (bleaching) [32] in the presence of high H_2_O_2_ concentration.

### Stability improvement by substituting oxidizable methionines

In a recent study, where VP was evolved for increased oxidative stability, methionine substitutions were not selected in the improved variant [33]. However, different authors have reported oxidative stability improvement after removal of methionine residues from a VP fusion protein [34], as well as from fungal MnP [35], GP [36] and dye-decolorizing peroxidase [37]. Given this background, and after verifying methionine oxidation in VP under oxidative conditions, a strategy was designed based on the replacement of methionines. This is the first time that all of them are simultaneously substituted in a heme peroxidase. The result was that the mutated variants at three or the four methionine residues behaved as the most stable ones, confirming the involvement of methionine oxidation in the inactivation process and, consequently, that our strategy has been sound.

We have also demonstrated that the native VP follows a time-dependent (with decreasing half-life values to increasing concentrations of H_2_O_2_) and saturation kinetic H_2_O_2_-mediated inactivation model, as reported for other heme peroxidases [32, 38–43]. In addition, our studies reveal that a positive cooperativity model contributes, at least in part, to the oxidative inactivation of VP by H_2_O_2_, in agreement with the sigmoidal profile characterizing the inactivation kinetics. Interestingly, this model is dependent on the oxidation of the methionine residues, since it is lost in the triple and quadruple methionine variants, where the cooperativity is not possible. According to this analysis, the substitution of three or four methionines by non-oxidizable residues abolishes this inactivation mechanism. In consequence, it can be considered that this is the reason of the stability improvement towards H_2_O_2_ in the M152F/M262F/M265L and M152F/M247F/M262F/M265L variants. The only negative impact lies in the enzymatic activity on the different substrates assayed, which was affected to different extents. At this respect, a correlation was found between *k*_cat_ and *k*_app_ values, both decreasing simultaneously in these variants (and also observed in the T45A/I103T and T45A/I103T/M152F/M262F/M265L mutants). Oxidative stability of heme peroxidases has been found to be the result of a competition between productive (reducing substrate) and unproductive electron sources (enzyme components including methionines) [29]. According to this idea, our results suggest that in the absence of reducing substrates the higher catalytic rate of native VP would result in a more rapid oxidation of methionines and other oxidizable residues leading to a faster inactivation compared with the mutated variants.

### Stability improvement by decreasing enzyme reactivity with H_2_O_2_

The second strategy aimed to improve the stability of VP towards H_2_O_2_ by the simultaneous substitution of Thr45 and Ile103 most probably perturbs the interaction between helices B and D. Mutations in the same protein region of the *Coprinopsis cinerea* peroxidase during directed evolution were related with a significant improvement in thermal and oxidative stability [36] after only minor structural changes [44]. Similarly, conformational changes due to a point mutation in the heme pocket has been also suggested to be responsible for the higher H_2_O_2_ resistance of a MnP variant [35].

In VP, the predicted structural changes associated to the T45A and I103T substitutions would affect the mobility and/or positioning of the conserved distal His47 and Arg43. These two residues, found in all functional peroxidases of the catalase-peroxidase superfamily, play a key role in the catalytic cycle [45] during resting enzyme activation and CI stabilization [46–48]. Stopped-flow spectrophotometry analysis of the T45A/I103T variant revealed that CI formation is impaired. The expected structural changes due to these two mutations led to a 2.8-fold decrease in the inactivation rate by H_2_O_2_, retaining the positive cooperativity inactivation model because of the presence of the four methionine residues. However, the decrease in the inactivation rate was not reflected in its half-life, being similar to that of the native enzyme.

### Final improvement by combining two stabilization strategies

The above two stabilization strategies were combined in the T45A/I103T/M152F/M262F/M265L variant. Only three of the four methionines present in VP were substituted in this variant since the replacement of the fourth one (Met247) did not provide any additional improvement. All the parameters evaluating the stability of the new enzyme towards H_2_O_2_ were improved, confirming the success of this final approach. The decrease in *k*_app_ was the result of the accumulative effect observed in the T45A/I103T and M152F/M262F/M265L variants, confirming that solving the oxidative instability problem of heme peroxidases has to be addressed using different (complementary) approaches. The T45A/I103T/M152F/M262F/M265L variant was by far the most stable VP under oxidative conditions. Its increased stability could be justified by the slowdown of three events that occur consecutively as deduced from the calculated velocities: i) CIII formation; ii) enzyme inactivation; and iii) heme bleaching. CIII is completely stable for circa 45 s in the presence of a 5000-fold stoichiometric excess of H_2_O_2._ This time should be enough to allow the enzyme to return to the RS in the presence of reducing substrates, minimizing the oxidative inactivation under operational conditions. The development of a mechanistic explanation for the improved peroxidase stability of this variant would be too speculative with the data currently available. However, taking into account that CIII is stabilized by complex interactions with residues of the heme distal side [45], we can hypothesize that changes at this environment due to the mutations introduced could modify these interactions increasing the CIII stability and reducing the bleaching rate of the enzyme.

In conclusion, the higher VP oxidative stability improvement obtained in this work has been achieved by substitution of amino acid residues located at specific positions near the heme group affecting formation, stabilization and decomposition of CIII. Additional changes at the heme distal side, together with the replacement of methionine residues, could further improve the stability of this enzyme towards H_2_O_2_. Moreover, the results presented here give insight into the strategy to be used to improve the oxidative stability of other peroxidases of biotechnological interest.

## Supporting Information

S1 Information Oxidative inactivation of native VP. (DOCX)

S2 Information Steady-state kinetics of the VP variants. (DOCX)
