# Supplementary material for: Improving the Oxidative Stability of a High Redox Potential Fungal Peroxidase by Rational Design
Source: PLoS One. 2015 Apr 29;10(4):e0124750. doi: 10.1371/journal.pone.0124750 (PMC4414599; doi:10.1371/journal.pone.0124750)
Supplement: S1 Information — (DOCX) [file pone.0124750.s001.docx]

**Supporting Information S1**

**Oxidative inactivation of native VP**

Native VP was incubated in the presence of increasing stoichiometric excesses of H_2_O_2_ at 4 ºC. The residual activity was measured with time and a progressive inactivation at all the H_2_O_2_:VP molar ratios assayed (from 500:1 to 40000:1) was observed, although some activity remained even when the enzyme was incubated for long periods of time. In this way, 43% of the initial activity remained after 25 min at a 1000:1 molar ratio and progressively decreased to reach a stable value of 12% after a few minutes at molar ratios between 10000:1 and 40000:1 (**S1 Fig.**). The loss of activity was complete at all the H_2_O_2_:VP ratios when the temperature of incubation was increased at 25 ºC (**S1 Fig.**, rhombs, includes an example of activity loss with 3000 H_2_O_2_ equivalents at this temperature).

The amino acid composition analysis of the inactivated enzyme at a 5000:1 H_2_O_2_:VP ratio, after 40 min at 25˚C, (**S2 Fig.**) revealed that cystine residues are oxidized to cysteic acid indicating disruption of disulphide bridges. Eight cysteine residues form four disulphide bridges stabilizing the enzyme molecular architecture [1], and their cleavage suggests global structural changes of VP under oxidative conditions. In the same way, the signal corresponding to methionine residues disappeared. Under these conditions, a new peak appeared in the HPLC chromatogram corresponding to methionine sulfone, confirming the oxidation of methionine residues.

**Reference for Supporting Information S1**

1. Pérez-Boada M, Ruiz-Dueñas FJ, Pogni R, Basosi R, Choinowski T, et al. Versatile peroxidase oxidation of high redox potential aromatic compounds: Site-directed mutagenesis, spectroscopic and crystallographic investigations of three long-range electron transfer pathways. J Mol Biol. 2005;354: 385-402.





**Figure S1.** **Oxidative stability of native VP.** Time course of the residual activity of native VP incubated at 4°C with different stoichiometric excesses of H_2_O_2_: 1000 (●), 2000 (○), 3000 (■), 5000 (□), 10000 (▲), 40000 (Δ) equivalents, and 3000 equivalents at 25°C (♦), estimated from Mn^2+^ oxidation in 0.1 M sodium tartrate, pH 5.0.


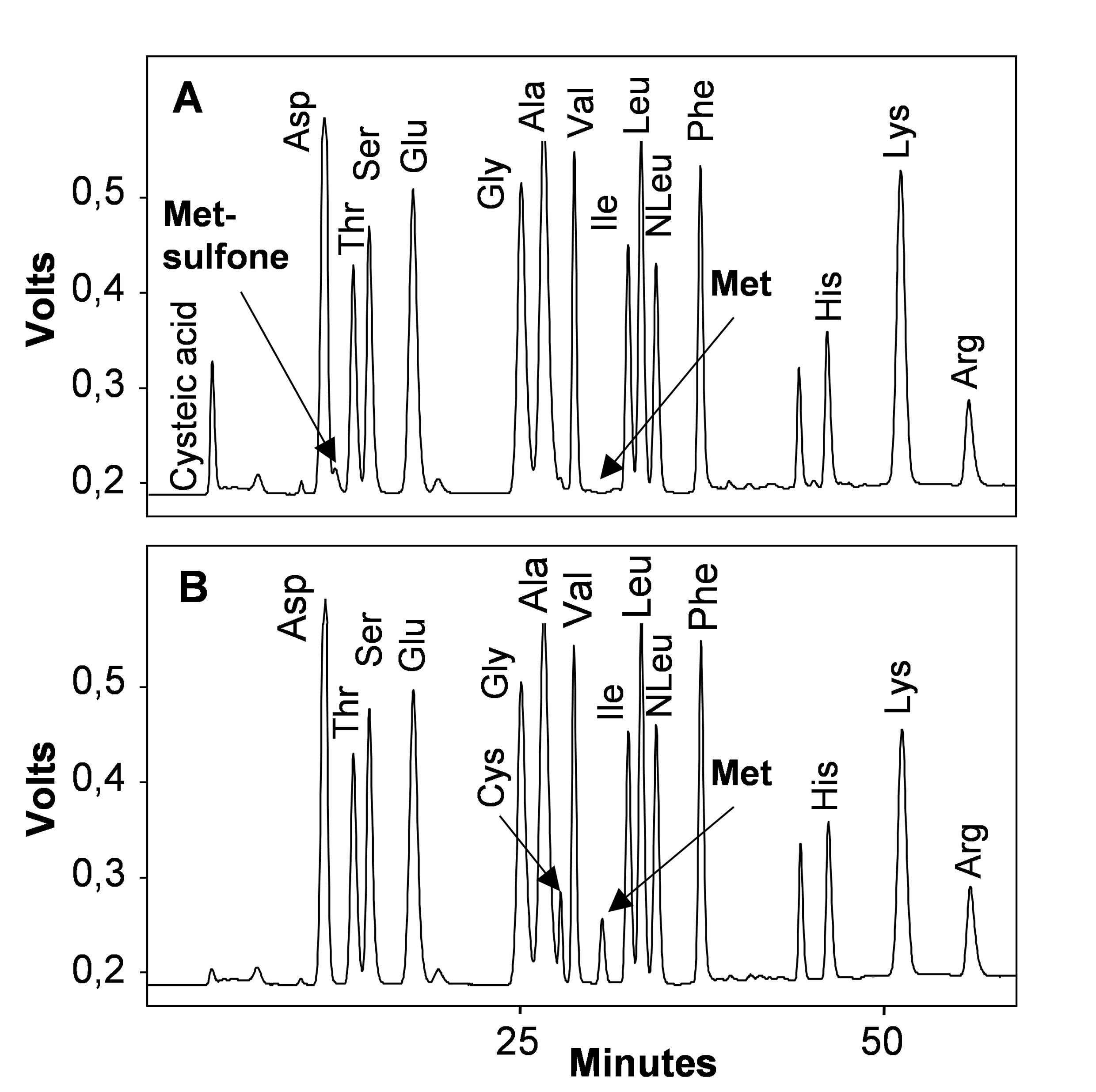


**Figure S2. Amino acid analysis of native VP.** Amino acid composition evaluated after enzyme incubation in the presence (**A**) and absence (**B**) of H_2_O_2_ (5000 equivalents). Peaks of methionine, methionine sulfone, cystine and cysteic acid are highlighted with arrows.
