# Supplementary material for: Improving the Oxidative Stability of a High Redox Potential Fungal Peroxidase by Rational Design
Source: PLoS One. 2015 Apr 29;10(4):e0124750. doi: 10.1371/journal.pone.0124750 (PMC4414599; doi:10.1371/journal.pone.0124750)
Supplement: S2 Information — (DOCX) [file pone.0124750.s002.docx]

**Supporting Information S2**

**Steady-state kinetics of** **the VP variants**

The steady-state kinetic constants for VA, Mn^2+^ and ABTS oxidation (at the exposed catalytic Trp164, Mn^2+^ oxidation site and main heme access channel, respectively), by the nine VP variants designed in this work, were determined (**S1 Table**) in order to investigate how the mutations affect the catalytic properties of this peroxidase.

In general, the single and double methionine variants exhibited similar catalytic efficiencies when compared with native VP. The M262F and M262F/M265L variants were the exception oxidizing ABTS, since they experienced a ~2-fold decrease in the catalytic efficiency for this substrate; together with the M247F variant, which was significantly impaired in its ability to oxidize the three substrates assayed (the efficiency values showing a 1.5 to 5-fold drop). Although these three variants exhibited a partial loss of activity on ABTS, the final reasons explaining this behaviour were different in the M247F, which suffered a 6-fold decrease in *k*_cat_, and the M262F and M262F/M265L variants, where a 1.7-1.8 fold increase in the *K*_m_ value was observed.

Regarding the M152F/M262F/M265L, M152F/M247F/M262F/M265L, T45A/I103T and T45A/I103T/M152F/M262F/M265L variants, all of them were affected in a similar way in their catalytic properties. The *k*_cat_ and *K*_m_ for VA oxidation suffered a decrease of 3.1 to 6-fold and 4.2 to 40-fold, respectively, resulting in mutated variants with an improvement in the catalytic efficiency as a consequence of the higher reduction in *K*_m_ (thereby increasing the affinity of the enzyme for this substrate) than in *k*_cat_. The M152F/M247F/M262F/M265L variant was the only of these enzymes that retained a similar catalytic efficiency on VA compared with the native enzyme because a similar reduction of ~4-5-fold was produced in both the *K*_m_ and *k*_cat_ values. Unlike what observed with VA, a decrease in the catalytic efficiency for oxidation of Mn^2+^ and ABTS by these four variants was produced: i) as a result of the decrease in *k*_cat_ and increase in *K*_m_, in the case of Mn^2+^ oxidation, with a higher *K*_m_ contribution (6.1-fold increment) in the T45A/I103T/M152F/M262F/M265L variant; and ii) due to a significant (2.6 to 6-fold) decrease in *k*_cat_ in the case of ABTS, without practically changes in *K*_m_ except in the M152F/M247F/M262F/M265L variant in which the value of this constant exhibited a 2-fold increase.

| **Table S1.** Steady-state kinetic constants [*K*_m_ (*µ*M), *k*_cat_ (s^-1^), and *k*_cat_/*K*_m_ (s^-1^·mM^-1^)] of native VP and variants for oxidation of VA, Mn^2+^, and ABTS in the low-efficiency site. | | | | | | |
| --- | --- | --- | --- | --- | --- | --- |
|  |  | VP | M152V | M247F | M262F | M265L |
| VA | *K*_m_ | 2600 ± 190 | 3400 ± 200 | 4100 ± 400 | 2500 ± 200 | 3900 ± 300 |
|  | *k*_cat_ | 5.8 ± 0.1 | 5.4 ± 0.1 | 4.3 ± 0.2 | 5.6 ± 0.1 | 11.4 ± 0.3 |
|  | *k*_cat_/*K*_m_ | 2.2 ± 0.1 | 1.6 ± 0.1 | 1.1 ± 0.1 | 2.3 ± 0.1 | 2.9 ± 0.2 |
| Mn^2+^ | *K*_m_ | 130 ± 11 | 119 ± 8 | 70 ± 5 | 162 ± 26 | 136 ± 7 |
|  | *k*_cat_ | 211 ± 4 | 219 ± 3 | 76 ± 1 | 233 ± 2 | 215 ± 2 |
|  | *k*_cat_/*K*_m_ | 1640 ±130 | 1840 ± 133 | 1090 ± 100 | 1440 ± 50 | 1580 ± 103 |
| ABTS | *K*_m_ | 1040 ± 80 | 1200 ± 740 | 869 ± 60 | 1740 ± 90 | 1490 ± 110 |
|  | *k*_cat_ | 209 ± 6 | 223 ± 5 | 35 ± 1 | 160 ± 4 | 224 ± 7 |
|  | *k*_cat_/*K*_m_ | 201 ± 10 | 186 ± 8 | 40 ±2 | 92 ± 3 | 151 ± 8 |
|  |  | M262F/M265L | M152F/  M262F/M265L | M152F/M247F/ M262F/M265L | T45A/I103T | T45A/I103T/M152F/  M262F/M265L |
| VA | *K*_m_ | 1900 ± 300 | 255 ± 28 | 622 ± 10 | 357 ± 30 | 65 ± 14 |
|  | *k*_cat_ | 3.6 ± 0.2 | 1.8 ± 0.1 | 1.1 ± 0.1 | 1.9 ± 0.1 | 0.35 ± 0.01 |
|  | *k*_cat_/*K*_m_ | 1.9 ± 0.2 | 7.0 ± 0.6 | 1.8 ± 0.2 | 5.2 ± 0.4 | 5.3 ± 1.0 |
| Mn^2+^ | *K*_m_ | 185 ± 12 | 255 ± 15 | 277 ± 28 | 371 ± 15 | 789 ± 51 |
|  | *k*_cat_ | 225 ± 3 | 147 ± 2 | 97 ± 2 | 158 ± 2 | 104 ± 2 |
|  | *k*_cat_/*K*_m_ | 1220 ±110 | 576 ± 34 | 350 ± 30 | 425 ± 15 | 132 ± 7 |
| ABTS | *K*_m_ | 1810 ± 90 | 1420 ± 110 | 1200 ± 129 | 932 ± 77 | 2030 ± 390 |
|  | *k*_cat_ | 186 ± 4 | 65 ± 2 | 35 ± 2 | 55 ± 2 | 79 ± 7 |
|  | *k*_cat_/*K*_m_ | 103 ± 3 | 46 ±2 | 29 ± 2 | 59 ± 3 | 39 ± 5 |
| Means and 95% confidence limits from reactions at 25 °C in 0.1 M tartrate (VA at pH 3, Mn^2+^ at pH 5 and ABTS at pH3.5). In native VP and some variants, a double kinetics for ABTS oxidation was observed [1] enabling calculation of a second set of constants (not shown) corresponding to a high catalytic efficiency site. | | | | | | |

**Reference for Supporting Information S2**

1. Morales M, Mate MJ, Romero A, Martínez MJ, Martínez AT, Ruiz-Dueñas FJ. Two oxidation sites for low redox-potential substrates: A directed mutagenesis, kinetic and crystallographic study on *Pleurotus eryngii* versatile peroxidase. J Biol Chem. 2012;287:41053-41067.
